# The significance of recruiting underrepresented minorities in medicine: an examination of the need for effective approaches used in admissions by higher education institutions

**DOI:** 10.3402/meo.v19.24891

**Published:** 2014-09-04

**Authors:** Obed Figueroa

**Affiliations:** Stony Brook University Graduate Alumni, Stony Brook, NY, USA

**Keywords:** underrepresented in medicine, minority recruitment, diversity, medical education

## Abstract

The purpose of this paper is to examine the significance of recruiting underrepresented minorities in medicine (URM). This would include African Americans, Hispanics, and Native Americans. The research findings support the belief that URMs, upon graduating, are more likely to become practitioners in underserved communities, thereby becoming a resource that prompts us to find effective ways to help increase their college enrollments statewide. This paper analyzes the recruitment challenges for institutions, followed by a review of creative and effective approaches used by organizations and universities. The results have shown positive outcomes averaging a 50% increase in minority enrollments and retention. In other areas, such as cognitive development, modest gains were achieved in programs that were shorter in duration. The results nevertheless indicated steps in the right direction inspiring further program developments.

‘If not us, then who? If not now, then when?’ President Obama credits Congressman John E. Lewis for carrying this question through his quests during the civil rights era (1). Whenever I read about the issues surrounding the shortage of US physicians and our health care needs, I think about Mr. Lewis's use of the famous quote. His words remind me that we have a responsibility to underrepresented minority students. My interest is to examine the areas of enrollment and retention of underrepresented minorities in medicine (URM) students going into medical education. I believe they represent the ‘who's’ mentioned by Lewis that can help address the health care disparities. This group might also help resolve the upcoming US physician shortage.

In 2011, there were 43,000 applicants applying to medical school in the United States. The American Association of Medical Colleges (AAMC) reported 20,176 applicants received letters of acceptance, resulting in 19,230 students matriculating into medical schools across the states (2). The report also reflected only 14.8% representation for underrepresented minority students (Appendices A and B). The college enrollment and practitioner numbers continue to remain significantly lower in minority groups in contrast to non-minority groups within medicine. In the medical industry, groups such as African Americans, Hispanics, and Native Americans are classified as URMs. This classification was established by the AAMC as a result of research evidence showing historically low representation of URMs within medical education. The data reported in the AAMC study, ‘Facts and Figures 2012’, show African American and Hispanic enrollment statewide in the single digits as compared to other groups (Appendix B).

The questions we should be asking as medical education professionals within industry are as follows:

1. What are the barriers and challenges for institutions and students?
2. What can be done to address the disparities?
3. Are the institutions of medical education providing adequate mentorship and guidance to students who are underrepresented in medicine? If so, what can we learn from them?
4. Are the schools of medicine, overall, concerned with the issues of minority enrollment?
5. Is there specific programming that can help inspire and motivate disadvantaged minority students’ interest in the areas of science?

These questions are significant and worthy of investigation. Institutions may not have the time to consider the value and impact of diversity within medicine. In addition, institutions will need to have skilled professionals in place who understand how to develop specific programming that addresses URM outreach, enrollment, and retention. Another institutional necessity will involve having someone in the leadership team that is skilled at finding funding resources that will help financially support the programming. Although these students are graduate students, many of them will enter into a world at a professional level, never having had similar exposure or adequate mentorship. Any successful institutional URM recruitment initiatives will require a holistic approach that is vested in creating campus environments that are challenging, motivating, and supportive to this cause.

## Literature review

### Challenges and barriers in recruiting URMs

In 2002, the American Medical Student Association (AMSA) task force and the Student National Medical Association (SNMA) conducted a study (AMSA-DS) which surveyed the challenges and barriers for colleges recruiting students that are classified as URMs. The study used qualitative methods to collect data which surveyed 144 medical colleges and universities, excluding Historical Black Colleges and Universities and schools in Puerto Rico, yielding a 59% response rate. The surveys were sent to both allopathic and osteopathic US medical schools statewide.

The focus for the AMSA diversity coalition was to research issues surrounding medical school admissions, in particular, URM recruitment and diversity, in general. The survey results identified 37 barriers for URM applicant/students (Appendix C). One of the identified areas showed results indicating that 90% of the surveyed colleges suggested that their URM applicants experienced low performances on the Medical College Admissions Test (MCAT) exam (3). This area, which has had an impact on minority recruitment, has been debated among students and scholars. What continues to be questioned is if the standardized exam has direct correlations to student success. From a student's perspective, they have resented the fact that one exam may be determining their career path. An AAMC national research study surveying 119 US medical school cohorts from 2001 to 2004 questioned the predictability of the MCAT exam (4). The results showed evidence that students who had lower MCAT scores also exhibited academic struggles. However, it was not the case for the majority of the cohorts (4). Furthermore, the study also suggested colleges to continue to use both the college grade point average (GPA) and the MCAT together in their admissions screenings in order to yield stronger correlations to academic performance and student persistence. Another area also ranked as a higher experience barrier was the URM undergraduate GPA and science preparation. The survey also assessed the sociocultural areas and found that there were challenges in communication skills and mentorship, as well as peer support. Other areas of significance causing access barriers were the financial and economic standing of disadvantaged URM applicants. The institutional survey results showed that 48% of the URMs experienced having financial concerns (3). The intimidation and lack of understanding about the college process may stretch back several generations for disadvantaged URMs. Breaking this cycle will require additional support systems, such as mentoring and study skills training in order to learn how to handle the rigors of graduate study.

### Effective recruitment solutions used by HEIs

#### The University of Chicago Pritzker School of Medicine

The University of Chicago Pritzker School of Medicine decided to implement a health disparity course as a recruitment aid to attract minority medical students. In addition, the AMSA decided to examine and evaluate the impact or influence of such courses on students that were classified as URM. The program entailed lectures; small group discussions; and site visits to local emergency rooms, hospitals, and community clinics. The curriculum topics included defining race, culture, ethnic bias, stereotypes, historical mistrusts, health literacy, language barriers, and health care systems. The students were placed in small groups, and in the end, they were required to conduct a presentation surrounding the issues of health care disparities.

There was 100% participation in the study because the surveying was integrated into the course. The administered survey was given to two entry classes from 2007 to 2008 and the average URM representation was 27%. The results showed that all surveyed groups, even those who were not minorities, personally experienced or knew someone who was directly affected by the impact of health care disparities. In addition, the survey identified that the URM students had a higher rate of work experience than other students within a socioeconomically diverse population prior to medical school (5).

The University of Chicago Pritzker School of Medicines began to see an impact of the health disparity course. Their URM enrollment continued to show an upward trend. Prior to the implementation of the course, the URM enrollment average was 11%. Two years after the course was implemented, the URM enrollment had doubled to 22%. The college acknowledges that there were other contributors to the URM enrollment efforts that helped to increase the percentages, but the health disparity course ranked the most influential. The other contributors having an impact on enrollment were college-sponsored events and outreach activities (5). The survey also identified that the health care disparity course was the highest ranked among students because it conveyed that the school was dedicated to health care disparities.

#### Partnerships

Another potential recruitment method used by universities is partnering with associations. The SNMA is a national organization established in 1964 and is under the auspices of the National Medical Association. The SNMA was created by students from Howard University and the Meharry College recognizing the need to give active support to minority medical students.

The SNMA local chapter at the University of Toledo College of Medicine (UTCOM) took the initiative to establish a formal collaboration with their university. This partnership's intent was to assist the university with its recruitment of students who are classified as URM. This approach focuses on utilizing a minority based student organization as a resource tool to achieve mutual recruitment goals. The establishment of the URM recruitment strategy was a collaborative effort between prospective students, current students, administrators, and faculty (6). The key to partnerships’ success largely depends on the institutions’ commitment and active participation.

The SNMA formulated a strategic initiative that outlined a recruitment plan listing dates, timelines, activities, and levels of participation. The SNMA also broke down their recruitment ideas into phases which provided the necessary structure for future reflection. The group evaluated each area of focus examining the challenges and solutions to be applied. It would be this framework that would help them establish a working model to guide their practice. Some of their findings influenced the following (6):

1. Establishing an SNMA chapter which was significant
2. Creating an institutional diversity committee
3. Formalizing a recruitment plan which incorporated partnerships
4. Integrating the SNMA chapter into the medical school interview process
5. Enhancing stronger communications with URM inquiries and applicants (6, pp. 1003–1004).

The partnership between UTCOM and the SNMA student organization has proven to have made a significant difference in the university medical school URM enrollment (Appendix D). The results yielded nearly a 50% difference within 1 year (6). In addition, the formation of this alliance will, in itself, continue to yield positive results as prospective students will perceive UTCOM actions as one that reflects an institution committed to diversity.

Duke University is a highly respected and top-ranked Ivy League institution. They, however, have not historically been successful in the area of producing PhD scholars of color. Spanning more than 30 years, the Biomedical and Engineering (BME) division's graduate program had never granted a PhD to an African American student. In 2004, the Duke's BME division awarded the third PhD to a Hispanic and its first to an African American (7). The challenge to increase their minority graduates students has not been limited to recruitment but also to the area of retention.

Dr. William M. Reichert, professor from Duke University, was presented with a leadership opportunity within Duke's BME division, where he was effective in implementing strategies that significantly increased the URM enrollment and retention at Duke University. Dr. Reichert acknowledges that the reason he was able to make the changes was a direct result of inheriting a position of authority. In his dual role as faculty and as the director of the URM fellowship program, he was able to construct a strategy which focused on building a unique team of science faculty that would assist him in increasing the URM enrollment in the biomedical fellowship program. Dr. Reichert was able to enroll 13 URM fellows and a team of 10 dedicated faculty members. Half of the students received NIH financial support. As a result, between 2000 and 2005 there was only one student that dropped out of the doctorate program. Dr. Reichert also pointed out that his grant funded program had a unique internal dynamic. He called it the ‘risk averse’ condition where the faculty role and position was directly linked to the students’ success (7). If the faculty did not help make the program work then it would fail, and so would the students. This dynamic, where tenure or job security does not apply indirectly acted as a motivator for faculty to find creative ways to be effective. He also deemed it quite significant to choose the right faculty for the program. He noted the following attributes as important when making the faculty selection:

1. Experienced and successful working with URM students
2. Having a vigorous research profile
3. Having the respect of their peers
4. Having control over resources for URM students
5. Being vested in student mentorship
6. Being dedicated to the mission (7).

Dr. Reichert identified the importance of creating a comfort zone for URM students. He encourages leadership to train faculty not to define the URM students by characteristics but rather by the nuances of the larger content (7). Dr. Reichert identified the importance of the staff remaining focused on the program's objectives. He did not want the faculty to carry stereotyped notions when they interacted with the URM students. Dr. Reichart cautions faculty to not focus on the students’ previous disadvantages. He also identified that the students must be encouraged to be receptive to mentorship from the majority faculty. Duke also offers an award winning summer research program for URMs and the disadvantaged. The program had an 80% success rate of students going on to other graduate programs or medical schools (7).

Dr. Reichart encourages faculty who are interested in addressing the issue of URM recruitment to start with where they have direct influence. Faculty can opt to mentor one student at a time or create a ‘home day’ where they can give a campus tour. Also, faculty can choose to influence admission committees by beginning a series of discussions surrounding the importance of campus diversity. Dr. Reichart's success has illustrated that victory is a collaborative effort that must have institutional support.

### Creative pipeline programming for URMs

The Medical College Admissions Test has historically been a challenge and barrier for disadvantaged and URMs. This standardized exam was designed to determine the mastery of the basic concepts of biology, chemistry, physics, verbal reasoning, and writing (8). Medical schools’ admissions committees use the MCAT exam as one of their screening tools to predict if an applicant will be able to perform academically within the medical school curriculum. Research has shown that this tool, as a screening mechanism in the admissions process, has had a negative impact on applicants from disadvantaged and URM groups. This has been counterproductive to the efforts of colleges and universities attempting to increase minority groups into the sciences (8).

The Indiana University School of Medicine decided to conduct a 4-year study in which they surveyed four cohorts during 2003–2006, entering into a 10-week intensive MCAT preparatory program. Their students were from disadvantaged and URM groups; 79% who were of African American descent. The ultimate goal of the program was to increase cognitive abilities that would improve the student's performance on the MCAT exam. The program requirements include a bachelor's degree, medical school science pre-requisites, and previous MCAT scores. All applicants are required to have a formal interview with a college representative. The program is full time and runs 5 days a week. The students are engaged in weekly lectures covering biology, general chemistry, organic chemistry, physics, and verbal reasoning.

The program also dedicates time with the students helping them to develop their writing skills. There are multiple factors that also influence the programs’ effectiveness. Students are engaged in a holistic view of learning and tutoring building positive attitudes and the acquisition of test taking skills (8). Another significant area with positive impact on the program is how it creates an integrated team consisting of instructors, students, and tutors. Students also participate in small learning groups led by first year medical students. The program also dedicates five sessions where the students take practice MCAT exams so they can become comfortable with the exam structure.

This study showed small but nevertheless cognitive gains (Appendix E). The participants on average raised their MCAT scores by three points (8). This would suggest facilitating a longer preparation program could yield continual upward shifts in the areas of cognitive development for students taking the MCAT exam. The study also showed an increase in non-cognitive areas such as the participant's self-confidence as well as an increase in ownership of their career trajectory.

Postbaccalaureate programs are another useful strategy for institutions seeking to create their own pipeline of students. Many have been successful in enriching students’ science foundation (11). Students who seek out this avenue are often interested in changing their careers, rejected in medical school interviews, or come from an organized lower level science programs. The University of Michigan offers a Post Bac program where their goal is to provide unsuccessful, non-traditional students with the necessary skills to succeed in upper level science programs and become physicians (9). The participants are students that are defined as economically disadvantaged and or URM. The curriculum provides the following:

1. Basic science courses
2. One year of research
3. Academic testing and counseling
4. Reading and study skills development
5. Mentoring and other supporting services (9).

The admission requirements for applicants are a minimum GPA of 2.0 and MCAT minimum mean scores of 4.5. Students that have previously enrolled in postbaccalaureate or a master's degree in the sciences are not eligible to enroll. The goal of the University of Michigan's postbaccalaureate program is to work with the students for 1 year in order to help develop their non-cognitive as well as cognitive abilities (9). Successful students complete the program with a GPA of 3.0 and an MCAT mean score of 6.5, which would create the opportunity for them to matriculate into the University of Michigan's medical school. The program also provides all participants with supportive services to assist with the transition. This would include helping students with the application and financial aid process. The program reports a high success rate of 80% matriculation entering into medical school programs (9).

Community-based pre-professional programs can also be an effective option for creating pipelines into medical school programs. Dr. Lynne Holden, a practicing physician of color who works in emergency medicine at Montefiore Medical Center, believes the way to address the health care disparities is to address the health care workforce (10). It was this fundamental belief that would inspire her in 2006 to establish ‘Mentoring in Medicine’. This is a community-based not-for-profit organization which focuses on facilitating specific programming designed to educate and inspire primarily disadvantaged African American and Hispanic students who are interested in the health sciences.

This strategic approach has three phases; recruitment, high school, and college/postbaccalaureate. These areas focus on reaching urban communities seeking to engage them in an opportunity that would expand their career awareness while providing educational platforms to build upon their abilities (10). More specifically, the three phases are as follows:

1. Recruitment – designing conferences and symposiums that educate the students and their family support systems
2. Creating pathways to health care careers: Creating specific curricula in high schools covering biology concepts, organ systems, disease, and introduction to health care concepts and health care pathways
3. College/postbaccalaureate programs: Providing mentorship and strategic planning for school admissions, test preparation, and internships (10).

The organization has helped more than 6,200 students and has received volunteer assistance from almost 600 professionals from the tri-state area (10). The achievements of the program have been largely due to the strategic planning, multiple collaborations, volunteers, donations, and grant funding.

## Conclusion

I would encourage our peers to reflect on their contributions to the efforts of URM recruitment. Institutions that have made efforts within their scope of practice have been successful. It is not only important to evaluate our belief system regarding URM recruitment but also that of our institutions. In order for recruitment initiatives to be effective and have longevity we require institutional supports. Research has shown that URM's contributions will help to meet the pending health care needs of our growing diverse society. Minority physicians are also more likely to practice in underserved communities (11). In addition, they are also more likely to choose primary care/family practice as a specialty. This contribution is significant and patients in urban communities are more receptive to physicians that are from their cultures (11).

As we examine ways to address the health care disparities in the United States, we must consider our own natural resources. We should continue to ask ourselves and others the difficult questions. Does progressive change require creative thinking to produce better outcomes? Should we reevaluate our admission screening practices? Could it be beneficial to humble ourselves and reach out to external resources in order to establish partnerships? Is it too late to reexamine if we have the right principles in place that can lead such initiatives?

The research findings in this paper have provided various techniques used by organizations and US higher education institutions. Their creative strategies have been shown to produce positive results and in some incidences double digit URM enrollment increases. It was my intent to provide examples of effective frameworks for consideration. As in any example, there may be areas that are not practical in your practice. However, any areas where institutions are reflective and proactive should yield positive results.

## Appendices

### (A) Figure 5. Number of U.S. Medical School Applicants by Race and Ethnicity, 1977–2011

Note: 1) White, Asian, Black, and Native American, are Non-Hispanic. Since 2002, individuals have the option of reporting both their race and ethnicity alone or in combination with some other race or ethnicity. In this figure, numbers are reported for race alone; 2) The data is adapted from by Castillo (2).

*From 1974–2001, includes Mexican American, Puerto Rican, and Other Hispanic. Since 2002, includes Cuban, Mexican American, Puerto Rican, Other Hispanic, and Multiple Hispanic.

Source: AAMC Data Warehouse: Applicant Matriculant File, as of 1/26/2012.

### (B) Figure 12. Percentage of U.S. Medical School Matriculants by Race and Ethnicity, 2010–2011

Note: 1) Those that reported more than one race are included under Non-Hispanic or more than One Latino Race; 2) The data is adapted from Castillo (2).

### (C) Barriers to Recruiting Minority Students

**Table 2:** Medical school reported barries to underrepresented minority recruitment

Barrier	Percent listing as barrier at their institution (%)
Legal/policy/regulatory	
Court decisions	33
State/local policies	19
State legislation limiting affirmative action	14
Educational	
Low MCAT scores	90
Low undergraduate GPA	60
Poor preparation in sciences	55
Absence of high school science interest programs	46
Low educational achievement	40
Lower quality of schools previously attended	34
Lower level of academic achievement among parents	30
Poor communication skills	19
No participation in service-oriented extracurricular activities	17
Socio-cultural	
Absence of role models	77
Lack of peer/community support	45
State/area population not diverse	37
Negative parental and cultural attitudes regarding careers	19
Financial/economic	
Lack of financial aid	48
Parental income level	39
Difficulties in finding financial resources for your school's programs	28
No financial travel assistance to the required admission interview	27
High application fees	14
Housing issues	12
Recruitment/admission	
Not enough minority faculty members	71
Other schools in the area targeting URM majoring in sciences	39
Absence of summer enrichment programs at your school	27
Race/ethnicity/gender composition of the admission committee	22
Absence of partnerships with private and public organizations	20
Lock of mentorship programs	20
Lack of career development outreach	16
No URM student recruiters	13
Complex application process	10
No pre-admission counseling and application assistance	7
Absence of an office of minority and/or multicultural affairs	7
No faculty member designated to address issues of concern from URM students	7

Note: The data is adapted from Agrawal et al. (3).

### (D)

**Table 1:** Underrepresented minority student matriculants at University of Toledo College of Medicine, 1996–2006

	1996	1997	1998	1999	2000	2001	2002	2003	2004	2005	2006
Blacks/African Americans	9	10	2	14	1	1	2	2	4	5	10
Hispanics (Mexicans included in this group)	3	2	8	6	2	3	2	1	1	1	4
Native Americans	0	0	1	0	1	0	1	1	2	2	0
Total URM	12	12	11	20	4	4	5	4	7	8	14
Total in class	135	134	135	139	140	143	151	154	135	145	145
% URM	8	9	8	14	3	5	3	2	5	5	9

Note: The data is adapted from Rumala and Cason (6).

### (E) Indiana School of Medicine – Summer MCAT program 2003–2006

**Table:** 3 Cohorts average cognitive gains

	A	B	C	D
Physical science	6.30	1.18	7.40	1.35
Verbal reasoning	6.30	1.45	6.90	1.68
Biological sciences	6.80	1.59	8.00	1.63
Total	19.30	3.14	23.30	3.54
Writing sample	5.60	1.72	5.80	1.59

A – Mean; B – Standard Deviation; C – Mean; D – Standard Deviation. Note: The data is adapted from Agbor-Baiyee (8).
